# Acute effects of phosphodiesterase 5 (PDE5) inhibition, ANP and NO on epididymal contractility are preserved despite chronic PDE5 exposure

**DOI:** 10.1186/1471-2210-11-S1-P47

**Published:** 2011-08-01

**Authors:** Andrea Mietens, Caroline Feuerstacke, Sabine Tasch, Gerrit Eichner, Ralph Theo Schermuly, Friedrich Grimminger, Dieter Müller, Ralf Middendorff

**Affiliations:** 1 Department of Anatomy and Cell Biology, Justus Liebig University, Giessen, Germany; 2 Mathematical Institute, Justus Liebig University, Giessen, Germany; 3 Department of Internal Medicine, Justus Liebig University, Giessen, Germany

## Background

To acquire motility and their fertilizing capacity, immotile spermatozoa have to transit from the testis through the epididymal duct to the distal parts of the organ where spermatozoa are stored until ejaculation. This transit mainly relies on contractions of the epididymal peritubular smooth muscle layer and various factors, including cGMP, contribute to its fine regulation [1,2]. Atrial natriuretic peptide (ANP) and nitric oxide (NO) affect contractility via cGMP-dependent pathways. Phosphodiesterases (PDEs) hydrolyzing cGMP control intracellular cGMP levels and thus limit a given cGMP signal. Sildenafil inhibits the cGMP-hydrolyzing PDE5 and thereby promotes relaxation of smooth muscle cells. Besides its use for the treatment of erectile dysfunction, sildenafil has gained importance as a therapeutic agent against pulmonary hypertension. Thus, an increasing number of young patients is exposed to chronic PDE5 inhibition. Currently, there is still very little knowledge about the occurrence and functional importance of PDEs in the epididymis and more specifically about possible effects or side effects of sildenafil on epididymal function.

## Materials and methods

RT-PCR combined with laser-assisted microdissection, Western blotting and organ bath studies were used to investigate occurrence and functional aspects of PDE5 in epidiymal tissue from man and rat.

## Results

Western blotting showed PDE5 expression in human epididymis. Immunohistochemistry together with RT-PCR analyses after laser capture microdissection localized PDE5 within the epididymal duct to smooth muscle cells, but not to epithelial cells. In organ bath studies with epididymal duct segments the cGMP-elevating agents ANP and NO resulted in a significant decrease of the frequency of spontaneous contractions. Sildenafil also significantly decreased spontaneous contractile frequency and its effect was additive to ANP and NO. However, after long-term exposure to sildenafil *in vivo*, spontaneous contractility of the epididymal duct was conserved as was the acute relaxing response towards ANP, NO and sildenafil. Expression of PDE5 remained unchanged.

## Conclusion

Data demonstrate that PDE5 is an important member of cGMP signalling pathways regulating the finely-orchestrated process of epididymal duct contractility and suggest, however, that in the epididymis side effects of therapeutically used sildenafil seem to be unlikely.
